# Surveillance of Drug Residue Profiles in *Gallus gallus domesticus* (Silkie Chickens) in Taiwan

**DOI:** 10.3390/ani14233529

**Published:** 2024-12-06

**Authors:** Chiao-Hsu Ke, Jr-Wei Chen, Chen-Si Lin

**Affiliations:** 1 Department of Veterinary Medicine, School of Veterinary Medicine, National Taiwan University, Taipei 10617, Taiwan; f08629002@ntu.edu.tw; 2 Poultry Industry Section, Department of Animal Industry, Ministry of Agriculture, Executive Yuan, Taipei 100212, Taiwan; li4653@moa.gov.tw; 3 Department of Animal Science and Technology, National Taiwan University, Taipei 10617, Taiwan

**Keywords:** veterinary drugs, antiprotozoal agents, veterinary epidemiology, veterinary drug residues, silkie chicken

## Abstract

Veterinary drugs are extensively utilized in poultry farming for purposes such as disease prevention, disease treatment, and feed efficiency enhancement. However, the application of these drugs can lead to unacceptable residues in edible products. This study aimed to investigate the residue profiles of veterinary drugs in silkie chickens. A total of 130 chicken samples were collected from two major retail markets in Taiwan between 2022 and 2024. The analysis of drug residues was conducted using liquid chromatography coupled with tandem mass spectrometry (LC–MS/MS). The overall detection rate of drug residues was 57.7%, and most of these residues were found to be below the maximum residue limits. Among the detected drugs, trimethoprim was the most prevalent, followed by nicarbazin, robenidine, decoquinate, diclazuril, and sulfamonomethoxine. Notably, there was a 31.4% chance that different samples from the same flock would yield varying results. Furthermore, a positive correlation was observed between drug residues and sample weight. In conclusion, this study provides valuable epidemiological data on drug residue profiles in silkie chickens in Taiwan. In the future, it is highly recommended that veterinary drug residues be continuously monitored, and food product sampling protocols be adjusted annually to ensure ongoing compliance with safety standards and protect consumer health.

## Simple Summary

Veterinary drugs are widely used in poultry farming. However, the overuse and/or misuse of veterinary drugs on farms poses severe threats to public health. This study aimed to investigate the residue profiles of veterinary drugs in silkie chickens, focusing on 48 veterinary drugs and 23 antiprotozoal agents. Liquid chromatography coupled with tandem mass spectrometry (LC–MS/MS) was employed to analyze drug residues. Among the investigated samples, almost all samples were compliant, with only a few cases exceeding the maximum residue limits based on regulations in Taiwan. Furthermore, different samples from the same sampling flock could produce inconsistent test results. There was also a positive correlation between drug residues and sample weight. The findings suggest that veterinary drug usage is generally appropriate, reflecting the commitment of both government authorities and farmers to maintain food safety. This study reports epidemiological data on drug residues in silkie chickens in Taiwan and provides possible directions for further studies.

## 1. Introduction

Veterinary drugs are widely used for disease prevention, treatment, and growth promotion in livestock production. These drugs, particularly antibiotics, play a crucial role in poultry farming, where they are used not only to treat and prevent disease but also to promote growth and improve feed efficiency [1]. The high stocking density in commercial poultry farming systems creates an environment conducive to disease transmission [2], making the use of these drugs essential for maintaining flock health [3]. However, the overuse and/or misuse of veterinary drugs on farms poses severe threats to public health. The residues of these drugs can remain in poultry meat, leading to potential health risks for consumers, including allergic reactions and the development of antimicrobial resistance [4,5]. The major causes of drug residue formation in poultry products have been linked to improper and illegal use, such as exceeding recommended dosages and inadequate withdrawal periods [6,7]. Despite regulations to monitor and limit veterinary drug residues, the global overuse of antibiotics in poultry remains a significant cause of antimicrobial resistance, which is recognized as a major public health threat [8].

As with antibiotics, the use of antiprotozoal drugs, particularly coccidiostats, is essential in the poultry industry to control coccidiosis. This disease can cause significant economic losses due to reduced weight gain, poor feed conversion, and high mortality in chickens [9]. Coccidiosis is particularly common in chickens raised under intensive farming conditions, particularly those in warm and humid environments. Factors such as overcrowding, substandard hygiene practices, and inadequate isolation of infected animals significantly facilitate the spread of this infection [10]. The infection can lead to severe intestinal damage, manifesting as lesions, diarrhea, poor feed efficiency, reduced weight gain, and death [11]. In poultry production, coccidiosis is one of the most economically damaging diseases due to its high prevalence and impact on growth performance [12]. To prevent the occurrence of coccidiosis, the prophylactic administration of coccidiostats is considered a financially efficient method of controlling coccidiosis in broiler chickens rather than therapeutic treatment [9]. However, the widespread use of coccidiostats has raised significant concerns regarding sanitation and public health because of potential drug resistance development. For example, the emergence of drug-resistant strains of *Eimeria*, which reduce the efficacy of these drugs, has been increasingly proposed [13]. These consequences have led to the increased use of multiple agents in combination to prevent treatment failure. This also indicates that the use of multiple antibiotics and antiprotozoal drugs has become widespread in the poultry industry, causing public health issues for customers. To address these risks, Taiwan’s regulatory agencies have established maximum residue limits (MRLs) for veterinary drugs in poultry products. Analytical techniques such as liquid chromatography–tandem mass spectrometry (LC–MS/MS) are also employed to monitor compliance and ensure food safety [8]. MRLs have been established for various veterinary drugs in several countries or regions, including Taiwan [14], and in some countries, the use of certain drugs as feed additives has been restricted or even banned [15].

In recent years, changes in dietary preferences have increased the consumption of colored broiler chickens, commonly known as silkie chickens. For example, a previous study indicated that elderly individuals and women prefer silkie chicken as a functional food to nourish their bodies [16]. In Taiwan, the unique flavor and nutritional value of silkie chicken have also contributed to its growing popularity in markets. Due to increased consumption, the importance of drug residues and food safety have also arisen. However, to the authors’ best knowledge, few studies have described the drug residue profiles, including the 48 veterinary drugs and 23 antiprotozoal drugs used in silkie chickens. Therefore, the aim of this study was to investigate the drug residue profiles of silkie chickens in Taiwan. The prevalences of several drug residue profiles are reported herein, with the aim of raising awareness of public health issues in the future.

## 2. Materials and Methods

### 2.1. Study Design

To detect drug residue profiles, a total of 130 silkie chicken samples were purchased from two major retail markets in Taiwan between April 2022 and January 2024. The samples were purchased and transported to the laboratory in collection containers with ice packs. About 200 to 300 g of chicken *pectoralis major* tissue were collected for each sample. The samples were chopped and kept frozen in sterile plastic bags at a set storage temperature (−20 °C). The experiments were performed three times on each sample. The determinations of 48 residual veterinary drugs [17,18] and 23 antiprotozoal agents were performed as previously described [14,19].

### 2.2. Multiresidue Analysis for Detection of 48 Residual Veterinary Drugs

#### 2.2.1. Chemicals and Reagents

Acetonitrile, methanol, and n-hexane (residual pesticide-PCB analysis grade), used for extraction, and LC/MS grade methanol and acetonitrile, employed in LC–MS/MS analyses, were purchased from Wako Pure Chemical Industries (Osaka, Japan). Ammonium acetate and acetic acid were also obtained from Wako Pure Chemical Industries (Osaka, Japan).

#### 2.2.2. Sample Preparation

Each sample was homogenized in an electric food processor and stored at −20 °C prior to analysis. Chicken *pectoralis major* tissue was used for fortification. A total of 1 mL of acetonitrile containing 5 to 50 ng/g of veterinary drugs and their metabolites was added to a 5 g portion of minced muscle tissue. This mixture was homogenized with 10 g of sodium sulfate and 25 mL of an acetonitrile–methanol solution (95/5, *v*/*v*) for 3 min at 10,000 rpm. After homogenization, the samples were centrifuged at 4500× *g* for 10 min at 4 °C. The supernatant was carefully decanted, and the pellet was re-homogenized with an additional 25 mL of the acetonitrile–methanol solution under the same conditions. The second homogenate was again centrifuged, and the supernatant was combined with the previous supernatant. The combined extract was washed with 30 mL of n-hexane saturated with acetonitrile, after which it was evaporated. The residue was then dissolved in 1.5 mL of methanol and prepared for analysis by LC–MS/MS.

#### 2.2.3. LC–MS/MS Analysis

A 10 µL aliquot of each sample or standard solution was injected into an ACQUITY UPLC^®^ HSS T3 (1.8 µm, 2.1 mm i.d. × 100 mm; Waters^TM^, Tosoh, Tokyo, Japan) column. The chromatographic elution was conducted at a flow rate of 0.3 mL/min at 35 °C using a mobile phase consisting of 10 mM ammonium acetate containing 0.3% acetic acid (solvent: water, phase A) and a mixture of acetonitrile–methanol (2:8, *v*/*v*) (phase B). The elution program started with 80% phase A for 2 min, followed by a linear gradient-reducing phase A from 80% to 5% over 8 min, and was maintained at 5% phase A for an additional 20 min. The veterinary drugs and their metabolites were quantified using an API2000 tandem mass spectrometer (Applied Biosystems, Foster, CA, USA), operated in both positive and negative ion modes. The ion spray voltage was set to 3.3 kV for the positive ion mode and −3.3 kV for the negative ion mode, with the interface vaporizer temperature fixed at 450 °C. The conditions of MS/MS (precursor ion, product ion, declustering potential, and collision energy) of each drug are listed in Table 1.

#### 2.2.4. Method Validation

Calibration curves were constructed using the daughter ion peak areas of the standard drugs spiked into extracts prepared from blank muscle samples. The detection limit was defined as a concentration yielding a signal three times the background noise, while the quantitation limit was determined as a signal ten times above the noise level. The calibration curves were constructed over a range of concentrations, and the correlation coefficients were calculated to assess the accuracy of the method. All the validation parameters of multiresidue analysis for the detection of 48 veterinary drugs are presented in Table 2. The specificity of the LC–MS/MS method for positive samples was demonstrated by the absence of any interfering peaks at the acquisition time in the samples (Figure S1).

### 2.3. Multiresidue Analysis for Detection of 23 Items

#### 2.3.1. Chemicals, Reagents, and Solution

Ultra-pure water (18.2 MΩ/cm) was obtained from a Millipore water purification system (Cork, Ireland). Acetonitrile (ACN), methanol (MeOH), and formic acid (FA) were purchased from Merck Ltd. (Darmstadt, Germany), while ammonium formate was supplied by Wako (Osaka, Japan) and Nacalai Tesque Inc. (Kyoto, Japan). Stock solutions of individual standards were prepared by dissolving 10 mg of each compound in 10 mL of the appropriate solvent (ACN, MeOH, or DMF), resulting in a concentration of 1 mg/mL. These stock solutions were stored in amber vials at −20 °C, where they remained stable for at least two months. Working solutions for spiking and calibration were prepared by further diluting the stock solutions with methanol.

#### 2.3.2. Sample Preparation

Homogenized samples (in 2.0 g aliquots) were placed in 50 mL polypropylene centrifuge tubes and spiked with standard solutions at 0.5, 1.0, and 10 mg/mL, as well as 10 mg/mL of stable isotope labeled (SIL) internal standard. After spiking, the samples were allowed to equilibrate at room temperature for 10 min. Following this, 10 mL of cooled water and 10 mL of the extraction solvent (95:5, ACN/MeOH with 1% FA) were added. The mixture was vortexed for 1 min at 1000 rpm in a Geno/Grinder 2010. Subsequently, QuEChERS powder (6 g of magnesium sulfate and 1.5 g of sodium citrate) was added, and the tube was vortexed again for 1 min before being centrifuged at 5000× *g* for 1 min. The supernatant was then transferred into 50 mL centrifuge tubes containing 10 mL of ACN-saturated n-hexane, vortexed for 1 min, and centrifuged again for 1 min at 5000× *g*. The supernatant was removed, and the clean-up procedure was repeated. The n-hexane layer was transferred into a 15 mL centrifuge tube and evaporated to dryness under nitrogen at 50 °C. The residue was reconstituted in 950 μL of 80% methanol containing 0.1% FA and filtered through a 0.22 μm PTFE membrane (Millipore, Cork, Ireland) prior to LC–MS/MS analysis.

#### 2.3.3. LC–MS/MS Analysis

Liquid chromatographic separation was performed with an ekspert™ ultraLC 100 ultra-performance liquid chromatograph (SCIEX, Framingham, MA, USA) equipped with an Agilent Poroshell 120SB-C18 column (2.7 µm, 3.0 mm × 150 mm). The column was maintained at 40 °C, and a gradient elution program was employed with a flow rate of 0.3 mL/min. The mobile phases consisted of 0.1% FA in 5 mmol/L ammonium formate (solvent: water, Phase A) and methanol with 0.1% FA (Phase B). The gradient started at 95:5 (A), where it was held for 1 min, and then it was increased to 100% B over 14 min before being held for 6 min. The total run time was 22 min and the injection volume was 10 μL. Mass spectrometric detection was carried out using a QTRAP 5500 system (SCIEX, Framingham, MA, USA) with both positive and negative electrospray ionization (ESI) modes. Ion spray voltages were set to 5.5 kV for the positive mode and −4.5 kV for the negative mode. The vaporizer temperature was set at 500 °C, with curtain gas, ion source gas 1, and ion source gas 2 pressures set at 20, 50, and 50 psi, respectively. The optimal multiple reaction monitoring (MRM) parameters for the target coccidiostats were the same as those in a previous study [14]. The conditions of MS/MS (precursor ion, product ion, declustering potential, and collision energy) of each drug are presented in Table 3.

#### 2.3.4. Method Validation

The method was validated by constructing standard calibration curves with concentrations ranging from 0.5 to 25.0 µg/L. These curves were prepared in 0.1% FA in 80% methanol, both as neat standards and as matrix-matched standards spiked into blank samples. The limit of detection (LOD) and limit of quantification (LOQ) were determined using signal-to-noise ratios of 3:1 and 10:1, respectively. The precision and accuracy of the method were assessed by spiking chicken muscle samples at three concentration levels (0.5, 1.0, and 10.0 mg/kg) in five replicates. All the validation parameters of multiresidue analysis for the detection of 48 veterinary drugs are presented in Table 4. The specificity of the LC–MS/MS method for positive samples was demonstrated by the absence of any interfering peaks at the drug retention time in the samples (Figure S1).

### 2.4. Statistical Analysis

The data are presented as mean ± SEM. Statistical analysis was conducted with the unpaired Student’s *t*-test or one-way ANOVA in GraphPad Prism software v10. A *p*-value < 0.05 was considered statistically significant.

## 3. Results

### 3.1. Drug Residue Profiles of Silkie Chickens in Taiwan

**Figure 1:**
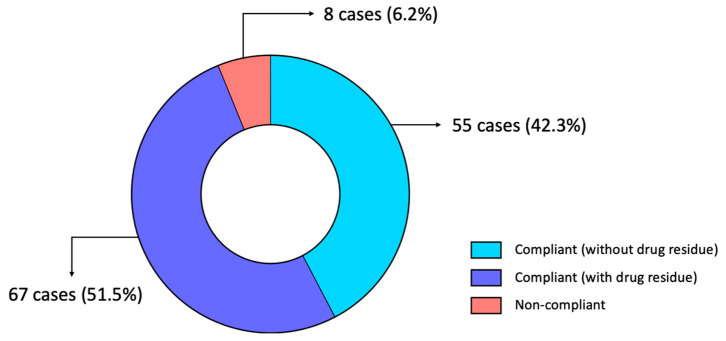
Percentage of compliant or non-compliant cases according to Taiwan’s regulations. A total of 130 cases were tested, including 122 compliant samples and eight non-compliant samples (red). In the 122 compliant samples, there were 55 cases without drug residues (light blue), and the resulting 67 were compliant cases with drug residues (dark blue).

To elucidate the drug residue profile of silkie chickens in Taiwan, 130 samples from two major retail markets were tested. Among these samples, 75 (57.7%) cases showed positive results. The remaining 55 cases showed negative results. In the positive samples (samples with drug residues detected), one type of drug was detected in 44 samples, followed by two, three, and four drugs detected in 18 samples, 12 samples, and 1 sample, respectively (Table 5). These results indicated that among all the samples, at least one drug residue was found in almost 60% of cases, highlighting a relatively high prevalence of drug residues in silkie chicken in Taiwanese retail markets. To further clarify the compliance status of the 75 samples with Taiwan’s regulations (Tables S1 and S2), we found that eight samples contained drug residues exceeding the MRLs, while the remaining 67 samples had drug residues, but the levels were below the MRLs. Therefore, as shown in Figure 1, 122 samples were classified as compliant (colored in bright and dark blue), whereas eight were deemed non-compliant (colored in red). Among the compliant 122 samples, the majority of cases had drug residues below the MRLs.

### 3.2. Percentages of Drug Residues in the Investigated Samples

**Figure 2:**
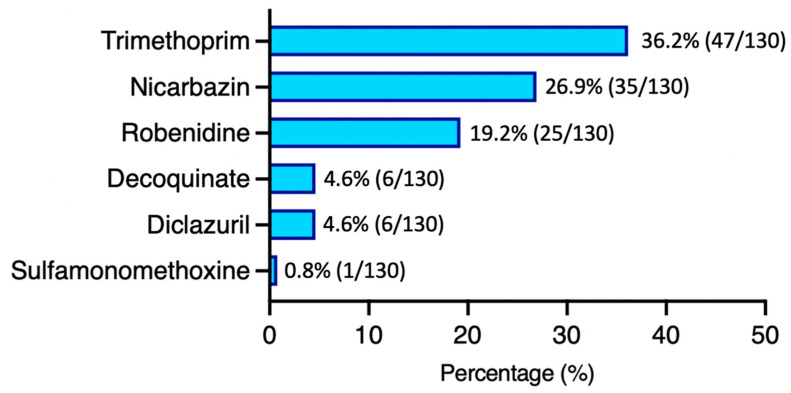
Percentage of drug residues in the investigated samples. The most common drug residue was trimethoprim (36.2%), followed by nicarbazin (26.9%), robenidine (19.2%), decoquinate (4.6%), diclazuril (4.6%), and sulfamonomethoxine (0.8%).

**Figure 3:**
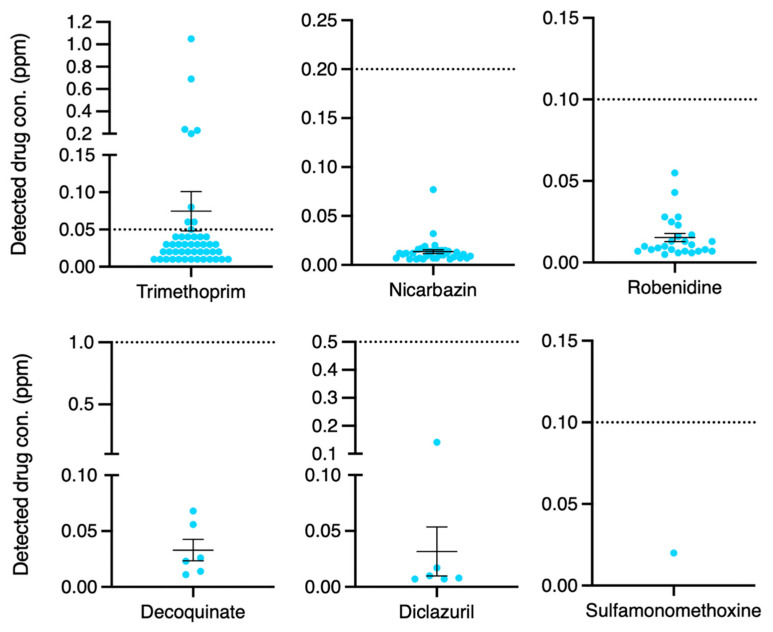
Distributions of drug concentrations in the drug-detected samples. The *X*-axis represents the tested drugs, and the *Y*-axis shows the detected concentrations (ppm). The dotted lines represent the maximum residue limits according to Taiwan’s regulations. Bars reflect mean ± SEM.

After analyzing the prevalence and compliance rates of drug residues, we further examined which drugs were most frequently detected. The most commonly detected drug residue was trimethoprim (47/130, 36.2%), followed by nicarbazin (35/130, 26.9%), robenidine (25/130, 19.2%), decoquinate (6/130, 4.6%), diclazuril (6/130, 4.6%), and sulfamonomethoxine (1/130, 0.8%; Figure 2). We also performed a distribution analysis of all detected drug residues, revealing that trimethoprim was the only substance found to exceed MRLs in eight samples (Figure 3). Notably, all the uncompliant samples (Figure 1) exhibited excessive trimethoprim residues. These results suggest that trimethoprim is not only the most frequently detected drug residue but also the only one found in violation of MRLs, highlighting the severity of its misuse in Taiwan’s poultry industry.

### 3.3. Consistency of Drug Residue Profiles in Randamly Seleted Flocks

**Figure 4:**
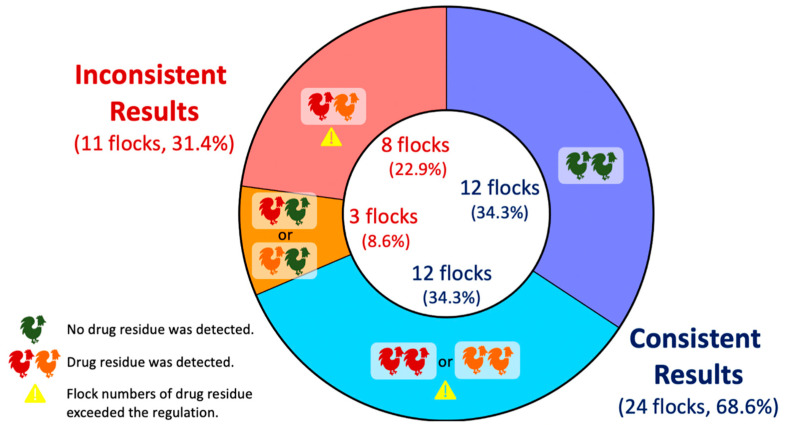
Comparisons of results from 35 randomly selected flocks with two cases simultaneously sampled. The blue parts of the donut figure represent the consistent results of two cases in the flocks, whereas the red and orange parts show the opposite results. The green chicken icon indicates that no drug residues were detected. The red and orange chicken icons show that drug residues were detected. The yellow warning icon represents the flock numbers of drug residues that exceeded the maximum residue limits.

Random sampling in a cyclic manner was employed, namely, collecting two samples, one sample, and then one sample in a flock again. Therefore, among all the investigated flocks, we conducted simultaneous sampling of two samples from 35 randomly selected flocks. The drug residue results from the two samples were then compared. We presumed that the drug residue profiles from these two samples might be consistent; however, as shown in Figure 4, among the 35 flocks, only 24 flocks exhibited consistent outcomes (68.6%). This included 12 flocks with no drug residues detected (34.3%, dark blue). The remaining 12 flocks had detectable drug residues (34.3%, light blue), including one flock providing two samples with trimethoprim residues exceeding the MRL. Notably, 11 flocks (31.4%) showed inconsistent drug residue profiles. Of these, three flocks had one sample with no drug residues detected, while the other one showed detectable residues (8.6%, orange). The other eight flocks had drug residues in both samples, but from different drugs. Furthermore, in one tested flock, only one sample exceeded the MRL, while the other was compliant (22.9%, red). These findings indicate that even within the same flock of silkie chickens, drug residue profiles can vary from sample to sample.

### 3.4. The Correlation Between Weight of Poultry Carcass and Drug Residues

**Figure 5:**
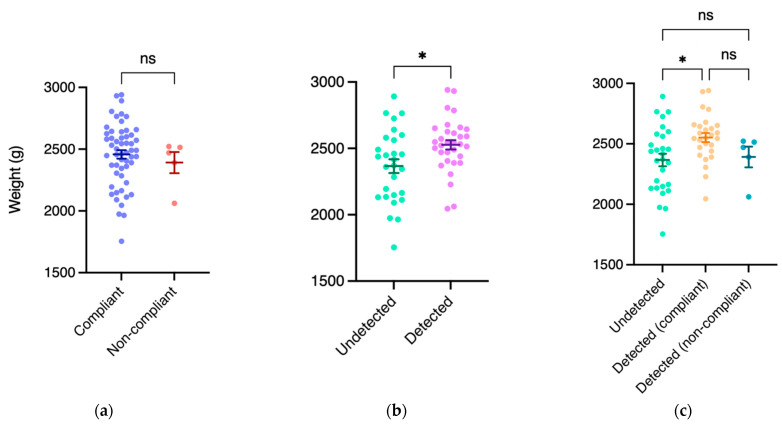
Comparisons of body weight of cases stratified by different parameters. (**a**) No significant difference was found in cases stratified by compliance; (**b**) cases without any drug detection exhibited significantly lower body weights; (**c**) cases with drug residues (below maximum residue limits) had elevated body weights compared with those without drug residues. Bars reflect mean ± SEM. Statistical analyses were performed with the unpaired Student’s *t*-test or one-way ANOVA. *—p < 0.05; ns—no significant difference.

To further clarify the correlation between poultry carcass weight and drug residues, we compared the sample weights from 60 randomly selected flocks. We found that there was no significant difference between compliant (2458.0 ± 255.4 g) and uncompliant samples (2392.0 ± 191.6 g, *p* = 0.5769, Figure 5a). However, the weights of samples with drug residues (2527.0 ± 201.3 g) were significantly higher than those of samples with no residues detected (2367.0 ± 275.5 g, *p* = 0.0119, Figure 5b). Subsequently, we then allocated the samples with drug residues based on compliance, categorizing them as undetected, detected (compliant), and detected (uncompliant). Samples with drug residues within MRLs (2552.0 ± 196.2 g) were significantly heavier than those with no residues detected (2367.0 ± 275.5 g, *p* = 0.0143); however, there was no significant difference in weight compared to the samples exceeding the MRLs (2392.0 ± 191.6 g, *p* = 0.3526, Figure 5c). These findings suggest that there might be a correlation between drug residues and the carcass weights of silkie chickens.

## 4. Discussion

Veterinary drugs are extensively employed for both the treatment and prevention of diseases in livestock. However, improper or excessive use of these drugs may result in residual traces in edible tissues, which potentially pose health risks to consumers. The current study reports the drug residue profiles of silkie chickens in Taiwan. In this study, samples with drug residues below the MRL comprised the largest group (51.5%), followed by those with no detection (42.3%), and those with relatively low MRL exceedance rates (6.2%, Figure 1). We also found that the most common drug with residues exceeding the MRL was trimethoprim, with a residue rate of 36.2% (Figure 2 and Figure 3). Furthermore, different samples from the same sampling flock had a 31.4% chance of yielding different test results (Figure 4). There was also a positive correlation between drug residues and sample weight (Figure 5). Taken together, this study reports epidemiological data on drug residues in silkie chickens in Taiwan and provides possible directions for further studies.

This study identified six drug residues, namely, trimethoprim (47/130, 36.2%), nicarbazin (35/130, 26.9%), robenidine (25/130, 19.2%), decoquinate (6/130, 4.6%), diclazuril (6/130, 4.6%), and sulfamonomethoxine (1/130, 0.8%). Trimethoprim, an antibiotic, showed the highest prevalence in the investigated silkie chicken samples. This prevalence (36.2%) is relatively high compared to findings in broiler chickens. A previous study from Vietnam found lower trimethoprim residue levels in broilers [20]. We speculated that the high prevalence resulted from the slow metabolism of trimethoprim in silkie chickens compared to that in broilers [21]. Furthermore, the distinct genomes of silkie chickens compared to other chicken populations [22] should also be taken into consideration. In addition to trimethoprim, this study identified several kinds of coccidiostats. Nicarbazin, an anticoccidial agent, had a prevalence of 26.9% in silkie chickens (Figure 2). Similar studies in broilers, however, reported lower prevalence rates. One survey reported a lower incidence of nicarbazin residues (6%) in 342 broilers [23]. Shen et al. also found a 9.45% nicarbazin residue rate in 277 samples [24]. In contrast, diclazuril residue exhibited a lower prevalence in this study; it was found in 4.6% of the silkie chickens. A previous study that investigated diclazuril residue showed a higher prevalence (11.9%) in broilers [24]. Based on our experience, we speculated that diclazuril is more rarely administered in the poultry industry than is nicarbazin, which potentially explained our findings. For robenidine and decoquinate, however, few epidemiological studies have reported drug residue rates in chickens. In the current study, we identified several antiprotozoal agents and speculated on the reasons for this phenomenon. First, we hypothesized that the main reason might be that silkie chickens are mostly floor-feeding. This might cause relatively severe coccidiosis, leading to the detection of different types of coccidiostat residues. Thus, to prevent drug resistance, the use of alternate coccidiostats in rotational programs would be needed, leading to diverse drug residues. Second, compared to that in broilers, the prevalence of drug residues in silkie chickens seems to be higher, possibly due to variations in farming practices and drug metabolism rates in this chicken strain. Though more evidence should be provided, we propose that the multiple drug residues might result from the severe coccidiosis in poultry farming and the unique traits of silkie chickens. Furthermore, the higher prevalences of trimethoprim and nicarbazin in silkie chickens also reflect a need for better drug management and/or regulations to ensure food safety.

This study also produced several interesting findings. Random sampling in a cyclic manner was employed, namely, collecting two samples, one sample, and then one sample in a flock again. Therefore, among all the investigated flocks, we conducted simultaneous sampling of two samples from 35 randomly selected flocks. We observed that different samples from the same flock could yield inconsistent drug residue profiles (Figure 4). This suggested not only individual variation among the samples but also emphasized the need for examining multiple samples from each flock for accurate representation of the status of drug residues. Therefore, in future studies, sampling more than two cases in the same flock is highly recommended. Furthermore, we found that samples with drug residues tended to have higher body weights compared to those without residues (Figure 5b). These findings revealed that the use of veterinary drugs might facilitate productivity in the poultry industry. These results also highlighted the importance of accurate drug administration, which not only enhances production efficiency but can ensure food safety for consumers.

## 5. Conclusions

In conclusion, this study investigated drug residue profiles in silkie chickens in Taiwan. Among the investigated samples, almost all samples were compliant, with only a few cases exceeding the MRLs based on regulations in Taiwan. Several coccidiostats were detected, implying the severity of coccidiosis in poultry farming. Notably, trimethoprim was not only the most frequently detected residue drug but the drug that most often exceeded MRLs. Furthermore, different samples from the same flock may show inconsistent residue levels. Overall, this study provides valuable epidemiological data on drug residues in silkie chickens in Taiwan. Although drug resides were identified, the MRL exceedance rate was relatively low. Our results highlight the need for further research to monitor certain drug residues to ensure food safety.

## Figures and Tables

**Table 1:** The parameters of multiresidue analysis for the detection of 48 veterinary drugs.

Analyte	Precursor Ion (*m*/*z*)	Product Ion (*m*/*z*)	Declustering Potential (V) ^1^	Collision Energy (eV) ^2^
Azaperol	330	121	30	30
		149	30	25
Azaperone	328	165	30	20
		121	30	20
Carazolol	299	116	30	20
		222	30	20
Ciprofloxacin	332	314	30	25
		231	30	45
Clopidol	192	101	45	25
		87	40	30
Danofloxacin	358	340	35	30
		283	40	25
Dicyclanil	191	150	30	25
		175	30	20
Difloxacin	400	356	35	20
		299	35	30
Enrofloxacin	360	316	35	20
		245	35	25
Eprinomectin	936.5	490	15	10
		352	15	10
Fleroxacin	370	326	30	20
		269	35	25
Flumequine	262	244	25	20
		202	25	30
Lomefloxacin	352	265	30	25
		308	30	15
Marbofloxacin	363	345	35	20
		72	30	25
Morantel	221	164	35	25
		149	35	35
Nalidixic acid	233	215	20	15
		187	20	25
Norfloxacin	320	302	30	20
		276	30	15
Ormetoprim	275	259	35	25
		123	35	25
Oxolinic acid	262	244	25	20
		216	25	35
Pefloxacin	334	316	30	20
		233	35	25
Pipemidic acid	304	217	30	20
		189	30	30
Piromidic acid	289	243	25	30
		271	25	20
Sarafloxacin	386	358	40	20
		342	35	20
Succinylsulfathiazole	356	256	35	15
		192	30	25
Sulfabenzamide	277	156	20	15
		92	20	30
Sulfacetamide	215	156	15	10
		92	15	25
Sulfachlorpyridazine	285	156	25	15
		92	20	30
Sulfadiazine	251	156	25	15
		92	25	25
Sulfadimethoxine	311	156	35	20
		92	30	35
Sulfadoxine	311	156	25	20
		92	30	30
Sulfaethoxypyridazine	295	156	30	20
		92	30	30
Sulfaguanidine	215	156	20	15
		92	25	25
Sulfamerazine	265	156	25	15
		92	25	30
Sulfameter	281	156	25	20
		92	30	30
Sulfamethazine	279	156	30	20
		186	30	15
Sulfamethizole	271	156	25	25
		92	25	25
Sulfamethoxazole	254	156	25	15
		92	25	25
Sulfamethoxypyridazine	281	156	25	15
		92	30	30
Sulfamonomethoxine	281	156	25	10
		92	30	30
Sulfapyridine	250	156	25	15
		92	30	30
Sulfaquinoxaline	301	156	25	15
		92	25	30
Sulfathiazole	256	156	25	15
		92	25	25
Sulfatroxazole	268	156	25	15
		92	25	30
Tetramisole	205	178	35	20
		123	25	30
Trichlorfon	259	109	20	20
		109	20	20
Trimethoprim	291	230	35	25
		123	35	25
Ethopabate	236	192	30	25
		132	30	35
Fluazuron	504	305	30	15
		307	30	15

^1^ Declustering potential is the difference between voltages at the orifice plate and skimmer. ^2^ Collision energy is the force of the current fragmentation.

**Table 2:** Validation parameters of multiresidue analysis for the detection of 48 veterinary drugs. LOQ—limit of quantification; RSD—relative standard deviation.

Analyte	Linearity (R^2^)	Calibration Range (ng/g)	LOQ (ng/g)	Precision (RSD%)		Recovery (%)
				Intraday	Interday	
Azaperol	0.9998	50–500	10	6.31	3.97	83.4
Azaperone	0.9994	50–500	10	2.80	9.62	81.0
Carazolol	0.9999	10–250	2	4.97	5.49	86.6
Ciprofloxacin	0.9997	50–500	10	5.04	3.19	73.5
Clopidol	0.9990	250–750	50	4.34	6.34	76.7
Danofloxacin	0.9998	50–500	10	0.36	1.06	82.3
Dicyclanil	0.9996	50–500	10	2.18	5.43	83.4
Difloxacin	0.9999	50–500	10	1.45	0.56	83.3
Enrofloxacin	0.9999	50–500	10	5.27	4.73	83.7
Eprinomectin	0.9998	50–500	10	2.56	6.62	73.4
Fleroxacin	0.9996	50–500	10	0.00	4.23	82.7
Flumequine	0.9988	50–500	10	3.30	3.00	83.1
Lomefloxacin	0.9997	50–500	10	2.29	4.50	73.4
Marbofloxacin	0.9999	50–500	10	4.24	2.62	81.9
Morantel	0.9999	50–500	10	3.27	3.27	80.8
Nalidixic acid	0.9999	50–500	10	1.07	9.02	84.0
Norfloxacin	0.9999	50–500	10	2.08	6.39	72.7
Ormetoprim	0.9996	250–750	50	4.72	4.18	80.6
Oxolinic acid	0.9998	50–500	10	1.87	2.19	91.7
Pefloxacin	0.9999	50–500	10	0.00	3.98	76.0
Pipemidic acid	0.9999	50–500	10	4.44	4.82	77.1
Piromidic acid	0.9998	50–500	10	2.22	0.95	73.0
Sarafloxacin	0.9991	25–500	5	1.32	7.79	76.3
Succinylsulfathiazole	0.9995	50–500	10	3.33	6.71	76.3
Sulfabenzamide	0.9994	50–500	10	1.37	0.82	87.1
Sulfacetamide	0.9997	50–500	10	2.03	1.50	79.6
Sulfachlorpyridazine	0.9989	100–500	20	1.15	1.17	86.7
Sulfadiazine	0.9995	50–500	10	0.63	3.04	95.2
Sulfadimethoxine	0.9996	50–500	10	5.00	4.75	100.4
Sulfadoxine	0.9997	50–500	10	0.23	2.75	87.4
Sulfaethoxypyridazine	0.9995	50–500	10	0.61	0.58	98.4
Sulfaguanidine	0.9998	50–500	10	3.29	3.46	77.2
Sulfamerazine	0.9997	50–500	10	0.53	3.64	94.3
Sulfameter	0.9983	50–500	10	3.86	0.11	99.1
Sulfamethazine	0.9999	50–500	10	2.24	6.28	90.1
Sulfamethizole	0.9999	50–500	10	5.32	0.00	92.6
Sulfamethoxazole	0.9998	50–500	10	2.81	4.44	90.1
Sulfamethoxypyridazine	0.9999	50–500	10	2.68	2.99	83.3
Sulfamonomethoxine	0.9997	50–500	10	1.44	0.46	84.0
Sulfapyridine	0.9995	50–500	10	0.75	0.41	105.9
Sulfaquinoxaline	0.9999	50–500	10	0.57	3.70	88.4
Sulfathiazole	0.9999	50–500	10	1.82	6.11	87.1
Sulfatroxazole	0.9997	50–500	10	3.66	3.76	91.7
Tetramisole	0.9999	50–500	10	1.25	1.90	80.7
Trichlorfon	0.9994	50–500	10	1.57	1.14	88.3
Trimethoprim	0.9997	50–500	10	2.60	3.42	81.7
Ethopabate	0.9998	50–500	10	2.12	4.90	88.8
Fluazuron	0.9992	250–750	50	3.48	7.92	84.8

**Table 3:** The parameters of multiresidue analysis for the detection of 23 coccidiostats.

Analyte	Precursor Ion (*m*/*z*)	Product Ion (*m*/*z*)	Declustering Potential (V) ^1^	Collision Energy (eV) ^2^
Buquinolate	362	148	58	50
	362	204		40
	362	260		22
Carnidazole	245	118	10	12
	245	75		30
	245	60		46
Decoquinate	418	372	64	20
	418	204		40
	418	232		34
Diaveridine	261	123	52	22
	261	245		26
	261	81		42
Diclazuril	405	334	20	18
	407	336		22
Dimetridazole	142	96	12	16
	142	81		22
Diminazene	142	120	20	5
	142	135		5
	282	120		5
	282	135		5
Halofuginone	416	100	24	20
	416	120		20
	416	138		20
HMMNI (2-Hydroxymethyl-1-methyl-5-nitro-1H-imidazole)	158	140	48	10
	158	55		16
	158	94		22
Imidocarb	349	188	36	24
	349	90		78
	349	162		22
Ipronidazole-OH	186	168	28	12
	186	122		20
	186	82		24
Isometamidium	460	313	4	18
	460	298		26
	460	269		46
2-Methyl-5- nitroimidazole	128	82	6	14
	128	56		12
	128	111		14
Metronidazole	172	128	20	14
	172	82		20
	172	111		20
Metronidazole-OH	188	123	28	12
	188	126		14
	188	144		12
Nicarbazine	301	137	36	20
	301	107		38
	301	46		48
Praziquantel	313	203	40	14
	313	174		26
	313	132		44
Pryrantel	207	150	24	26
	207	136		26
	207	97		22
Pyrimethamine	249	177	20	26
	249	198		38
	249	233		26
Robenidine hydrochloride	334	111	52	42
	334	138		24
	334	155		18
Ronidazole	201	140	24	12
	201	55		20
Tinidazole	248	121	15	17
	248	82		25
Zoalene	224	181	10	10
	224	77		24
	224	151		16

^1^ Declustering potential is the difference between voltages at the orifice plate and skimmer. ^2^ Collision energy is the force of the current fragmentation.

**Table 4:** Validation parameters of multiresidue analysis for the detection of 23 coccidiostats. LOQ—limit of quantification; RSD—relative standard deviation.

Analyte	Linearity (R)	Calibration Range (ng/g)	LOQ (ng/g)	Precision (RSD%)		Recovery (%)
				Intraday	Interday	
Buquinolate	0.9997	5–100	5	4.63	0.99	101.2
Carnidazole	0.9993	5–100	5	1.20	3.16	103.0
Decoquinate	0.9994	5–100	5	1.42	0.61	98.0
Diaveridine	0.9985	5–100	5	0.20	9.74	109.8
Diclazuril	0.9999	5–100	5	4.07	1.14	106.0
Dimetridazole	0.9992	5–100	5	1.98	2.48	98.0
Diminazene	0.9994	5–100	5	1.96	4.02	101.6
Halofuginone	0.9993	5–100	5	2.66	4.02	106.6
HMMNI (2-Hydroxymethyl-1-methyl-5-nitro-1H-imidazole)	0.9983	10–100	10	8.03	3.39	110.2
Imidocarb	0.9983	5–100	5	1.43	2.56	100.4
Ipronidazole-OH	0.9989	5–100	5	2.82	1.95	101.4
Isometamidium	0.9985	5–100	5	5.41	8.20	106.6
2-Methyl-5-nitroimidazole	0.9984	10–100	10	5.40	8.76	104.8
Metronidazole	0.9997	5–100	5	4.78	1.89	106.6
Metronidazole-OH	0.9994	5–100	5	4.07	3.93	104.8
Nicarbazine	0.9990	5–100	5	9.76	1.16	104.2
Praziquantel	0.9995	5–100	5	0.79	2.55	103.2
Pryrantel	0.9998	5–100	5	5.13	2.83	104.4
Pyrimethamine	0.9994	5–100	5	2.12	3.58	104.2
Robenidine hydrochloride	0.9993	5–100	5	2.90	1.15	104.2
Ronidazole	0.9987	5–100	5	4.63	3.78	93.4
Tinidazole	0.9998	5–100	5	1.98	0.87	92.8
Zoalene	0.9995	5–100	5	5.16	5.81	100.2

**Table 5:** Results of drug residues in 130 silkie chickens.

Results	Case Number (Percentage)
**Detected (positive)**	**75 (57.7%)**
One of the drugs detected	44 (33.8%)
Two of the drugs detected	18 (13.8%)
Three of the drugs detected	12 (9.2%)
Four of the drugs detected	1 (0.8%)
**Undetected (negative)**	**55 (42.3%)**

## Data Availability

The data presented in this study are available on request from the corresponding author.

## Supplementary Materials

The following supporting information can be downloaded at https://www.mdpi.com/article/10.3390/ani14233529/s1, Table S1: The maximum residue limit for the detection of 48 veterinary drugs in chicken muscles; Table S2: The maximum residue limit for the detection of 23 coccidiostats in chicken muscles; Figure S1: Representative chromatograms of positive samples.
