# Supplementary material for: Surveillance of Drug Residue Profiles in Gallus gallus domesticus (Silkie Chickens) in Taiwan
Source: Animals (Basel). 2024 Dec 6;14(23):3529. doi: 10.3390/ani14233529 (PMC11639894; doi:10.3390/ani14233529)

animals

MDPI

Table S1. The maximum residue limit for the detection of 48 veterinary drugs in chicken muscles. -, shall not be detected.

Analyte	Maximum residue limit (ppm)
Azaperol	-
Azaperone	-
Carazolol	-
Ciprofloxacin	-
Clopidol	5
Danofloxacin	0.2
Dicyclanil	-
Difloxacin	-
Enrofloxacin	0.1
Eprinomectin	-
Fleroxacin	-
Flumequine	0.5
Lomefloxacin	-
Marbofloxacin	-
Morantel	-
Nalidixic acid	-
Norfloxacin	-
Ormetoprim	0.1
Oxolinic acid	0.1
Pefloxacin	-
Pipemidic acid	-
Piromidic acid	-
Sarafloxacin	0.01
Succinylsulfathiazole	-
Sulfabenzamide	0.1
Sulfacetamide	0.1
Sulfachlorpyridazine	0.1
Sulfadiazine	0.1
Sulfadimethoxine	0.1
Sulfadoxine	0.1
Sulfaethoxypyridazine	0.1
Sulfaguanidine	0.1
Sulfamerazine	0.1
Sulfameter	0.1
Sulfamethazine	0.1
Sulfamethizole	0.1
Sulfamethoxazole	0.1
Sulfamethoxypyridazine	0.1
Sulfamonomethoxine	0.1
Sulfapyridine	0.1
Sulfaquinoxaline	0.1
Sulfathiazole	0.1
Sulfatroxazole	0.1
Tetramisole	0.01
Trichlorfon	-
Trimethoprim	0.05

Ethopabate	0.5
Fluazuron	-

Table S2. The maximum residue limit for the detection of 23 coccidiostats in chicken muscles. -, shall not be detected.

Analyte	Maximum residue limit (ppm)
Buquinolate	0.1
Carnidazole	-
Decoquinate	1
Diaveridine	0.05
Diclazuril	0.5
Dimetridazole	-
Diminazene	-
Halofuginone	0.1
HMMNI (2-Hydroxymethyl-1-methyl-5-nitro-1H-imidazole)	-
Imidocarb	-
Ipronidazole-OH	-
Isometamidium	-
2-Methyl-5- nitroimidazole	-
Metronidazole	-
Metronidazole-OH	-
Nicarbazine	0.2
Praziquantel	-
Pryrantel	-
Pyrimethamine	0.05
Robenidine hydrochloride	0.1
Ronidazole	-
Tinidazole	-
Zoalene	3

Figure S1. Representative chromatograms of positive samples. MRM, multiple reaction monitoring; ppb, parts per billion; RT, retention time; SMM, sulfamonomethoxine; TMP, trimethoprim.

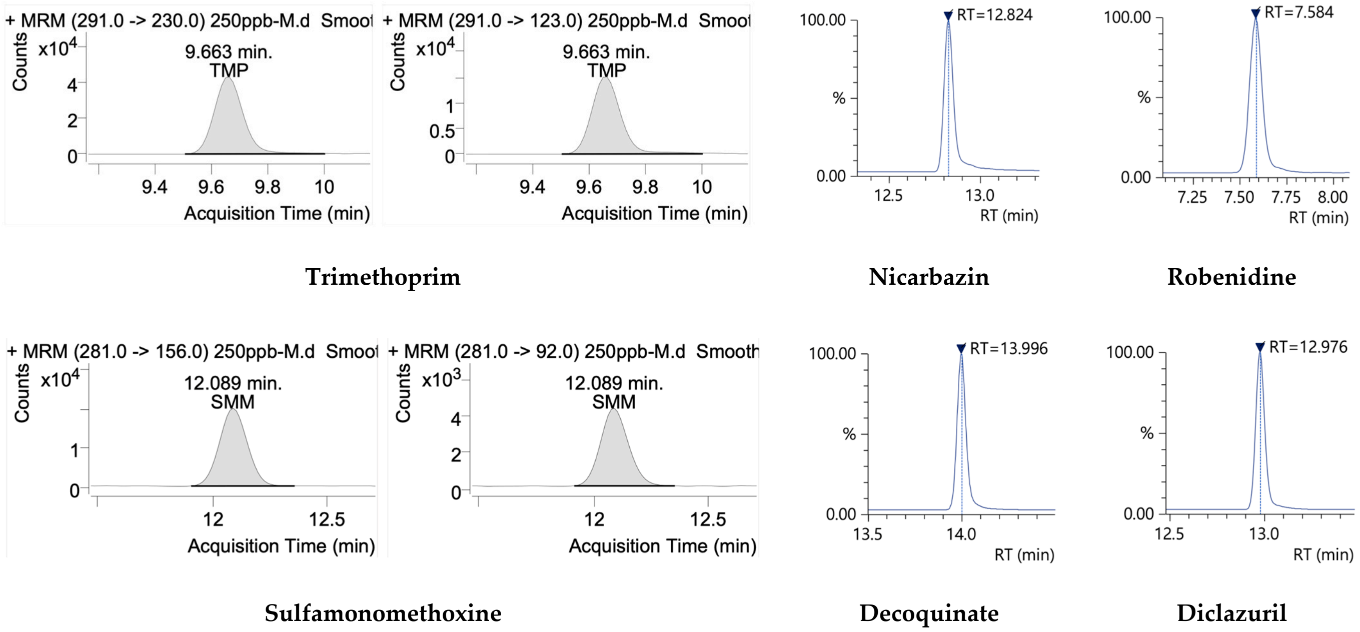

Supplement: Supplementary file 1 [file animals-14-03529-s001.zip › animals-3294340-supplementary.pdf]
